# *N*,*N*-Diethyl-2-hy­droxy­ethanaminium 5-(5-chloro-2,4-dinitro­phen­yl)-2,6-dioxo-1,2,3,6-tetra­hydro­pyrimidin-4-olate hemihydrate

**DOI:** 10.1107/S1600536812003248

**Published:** 2012-01-31

**Authors:** Rajamanickam Babykala, Doraisamyraja Kalaivani

**Affiliations:** a Postgraduate and Research Department of Chemistry, Seethalakshmi Ramaswami College, Tiruchirappalli 620 002, Tamil Nadu, India

## Abstract

The asymmetric unit of the title salt, C_6_H_16_NO^+^·C_10_H_4_ClN_4_O_7_^−^·0.5H_2_O, contains two cations, two anions and one water mol­ecule. In one independent anion, one nitro group is rotationally disordered over two orientations in a 0.657 (8):0.343 (8) ratio. In the crystal, inter­molecular N—H⋯O and O—H⋯O hydrogen bonds link all the components into ribbons extending along [100].

## Related literature

For details of the pharmacological properties of pyrimidine derivatives, see: Hueso *et al.* (2003 ▶); Colorado & Brodbelt (1996 ▶); Kalaivani *et al.* (2008 ▶); Kalaivani & Buvaneswari (2010 ▶). For the crystal structures of related compounds, see: Kalaivani & Malarvizhi (2009 ▶); Buvaneswari & Kalaivani (2011 ▶).
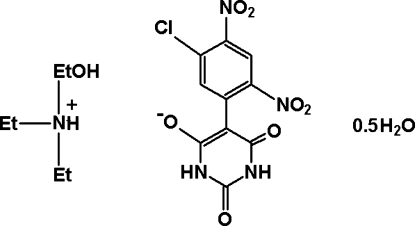


## Experimental

### 

#### Crystal data

C_6_H_16_NO^+^·C_10_H_4_ClN_4_O_7_^−^·0.5H_2_O
*M* *_r_* = 454.83
Triclinic, 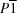

*a* = 12.3775 (7) Å
*b* = 12.8109 (8) Å
*c* = 13.5834 (9) Å
α = 101.497 (4)°
β = 99.142 (3)°
γ = 99.718 (3)°
*V* = 2038.6 (2) Å^3^
*Z* = 4
Mo *K*α radiation
μ = 0.25 mm^−1^
*T* = 294 K
0.30 × 0.25 × 0.20 mm

#### Data collection

Bruker Kappa APEXII CCD diffractometer
Absorption correction: multi-scan (*SADABS*; Bruker, 2004 ▶) *T*_min_ = 0.901, *T*_max_ = 0.942
36654 measured reflections
7179 independent reflections
5639 reflections with *I* > 2σ(*I*)
*R*_int_ = 0.031

#### Refinement

*R*[*F* ^2^ > 2σ(*F* ^2^)] = 0.043
*wR*(*F* ^2^) = 0.138
*S* = 1.07
7179 reflections
598 parameters
4 restraints
H atoms treated by a mixture of independent and constrained refinement
Δρ_max_ = 0.72 e Å^−3^
Δρ_min_ = −0.32 e Å^−3^

### 

Data collection: *APEX2* (Bruker, 2004 ▶); cell refinement: *APEX2* and *SAINT-Plus* (Bruker, 2004 ▶); data reduction: *SAINT-Plus* and *XPREP* (Bruker, 2004 ▶); program(s) used to solve structure: *SIR92* (Altomare *et al.*, 1993 ▶); program(s) used to refine structure: *SHELXL97* (Sheldrick, 2008 ▶); molecular graphics: *ORTEP-3* (Farrugia, 1997 ▶) and *Mercury* (Macrae *et al.*, 2008 ▶); software used to prepare material for publication: *SHELXL97*.

## Supplementary Material

Crystal structure: contains datablock(s) global, I. DOI: 10.1107/S1600536812003248/cv5228sup1.cif

Structure factors: contains datablock(s) I. DOI: 10.1107/S1600536812003248/cv5228Isup2.hkl

Supplementary material file. DOI: 10.1107/S1600536812003248/cv5228Isup3.cml

Additional supplementary materials:  crystallographic information; 3D view; checkCIF report

## Figures and Tables

**Table 1:** Hydrogen-bond geometry (Å, °)

*D*—H⋯*A*	*D*—H	H⋯*A*	*D*⋯*A*	*D*—H⋯*A*
N5—H5⋯O9^i^	0.91	1.85	2.749 (2)	167
O8—H8⋯O1^ii^	0.82	1.98	2.674 (2)	141
O9—H9*A*⋯O2	0.84 (1)	1.85 (1)	2.691 (2)	171 (3)
O9—H9*B*⋯O10	0.85 (1)	2.11 (2)	2.850 (2)	146 (3)
N2—H2⋯O3^ii^	0.85 (3)	1.99 (3)	2.826 (2)	168 (2)
N1—H1⋯O10	0.84 (2)	2.10 (2)	2.933 (2)	171 (2)
N10—H10⋯O12	0.91	1.85	2.698 (2)	154
O17—H17⋯O8^iii^	0.82	1.91	2.667 (2)	153
N7—H7⋯O17	0.84 (3)	2.07 (3)	2.911 (2)	176 (2)
N6—H6*A*⋯O1	0.81 (2)	2.16 (2)	2.947 (2)	167 (2)

Symmetry codes: (i) 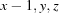; (ii) 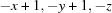; (iii) 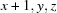.

**Fig. 1.**
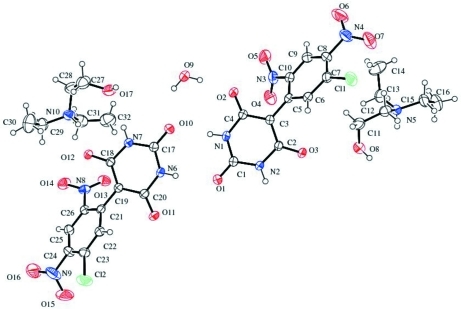
The content of asymmetric part of (I) showing the atomic numbering and 30% probability displacement ellipsoids. For the disordered nitro group, only major part is shown.

**Fig. 2.**
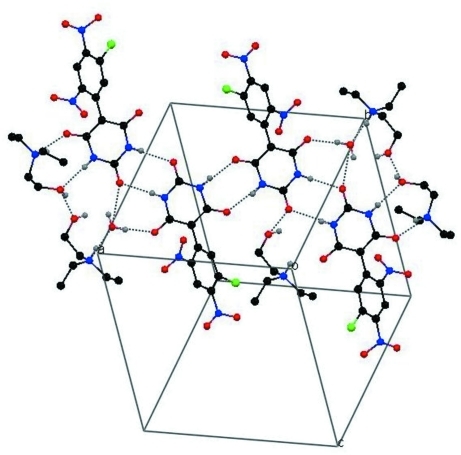
A portion of the crystal packing showing hydrogen bonds as dotted lines.

## supplementary crystallographic information

### Comment

Pyrimidine derivatives play a significant role in many biological systems (Hueso *et al.*, 2003). Barbiturates are pyrimidine derivatives which have been used as hypnotic drugs, anaesthetics, sleeping agents and for the treatment of anxiety, epilepsy and other psychiatric disorders (Colorado & Brodbelt, 1996). In continuation of our systematic studies of molecular salts containing various derivatives of barbiturates (Kalaivani *et al.*, 2008; Kalaivani & Malarvizhi, 2009; Kalaivani & Buvaneswari, 2010; Buvaneswari & Kalaivani, 2011), we present here the title compound (I).

In (I) (Fig. 1), all bond lengths and angles are normal and comparable with those observed in the related compounds (Kalaivani & Malarvizhi, 2009; Buvaneswari & Kalaivani, 2011). The crystal packing (Fig. 2) exhibits a number of N—H···O and O—H···O hydrogen bonds with various ring motifs such as *R*_2_^2^(8), *R*_2_^2^(9), *R*_3_^2^(8) and *R*_4_^4^(13), respectively. These hydrogen bonds link all moieties into ribbons extended in [100].

### Experimental

Analytical grade 1,3-dichloro-4,6-dinitrobenzene (DCDNB) and barbituric acid were used as supplied by Aldrich company. *N*,*N*-Diethylethanolamine was distilled under reduced pressure and the fraction boiling over at its boiling point was used for the preparation of the title molecular salt. DCDNB (2.01 g, 0.01 mol) in 15 ml absolute ethanol was mixed with barbituric acid (1.28 g, 0.01 mol) in 30 ml of absolute ethanol. *N*,*N*-diethylethanolamine (2.36 g, 0.02 mol) was added to the above mixture at 313 K and shaken well for 5–6 h. The solution was filtered and kept as such at room temperature for 48 h. On standing, reddish orange crystals come out from the solution. The crystals were powdered well and washed with copious amount of ethanol and dry ether and recrystallized from absolute alcohol (yield of pure crystals 80%, m.p. 503 K). Good quality crystals (red orange blocks) for single-crystal X-ray studies were obtained by slow evaporation of ethanol at room temperature.

### Refinement

The H atoms of the water molecule (H9A and H9B) and pyrimidine N atoms (H1, H2, H6A and H7) were located in difference Fourier maps and refined as restrained and riding in their as-found relative positions. The rest H atoms were positioned geometrically and were refined using a riding model.

### Crystal data

**Table:**

C_6_H_16_NO^+^·C_10_H_4_ClN_4_O_7_^−^·0.5H_2_O	*Z* = 4
*M**_r_* = 454.83	*F*(000) = 948
Triclinic, *P*1	*D*_x_ = 1.482 Mg m^−^^3^
Hall symbol: -P 1	Mo *K*α radiation, λ = 0.71073 Å
*a* = 12.3775 (7) Å	Cell parameters from 6078 reflections
*b* = 12.8109 (8) Å	θ = 2.5–27.0°
*c* = 13.5834 (9) Å	µ = 0.25 mm^−^^1^
α = 101.497 (4)°	*T* = 294 K
β = 99.142 (3)°	Block, red
γ = 99.718 (3)°	0.30 × 0.25 × 0.20 mm
*V* = 2038.6 (2) Å^3^	

### Data collection

**Table:**

Bruker Kappa APEXII CCD diffractometer	7179 independent reflections
Radiation source: fine-focus sealed tube	5639 reflections with *I* > 2σ(*I*)
graphite	*R*_int_ = 0.031
ω and φ scans	θ_max_ = 25.0°, θ_min_ = 1.7°
Absorption correction: multi-scan (*SADABS*; Bruker, 2004)	*h* = −14→14
*T*_min_ = 0.901, *T*_max_ = 0.942	*k* = −15→15
36654 measured reflections	*l* = −16→16

### Refinement

**Table:**

Refinement on *F*^2^	Primary atom site location: structure-invariant direct methods
Least-squares matrix: full	Secondary atom site location: difference Fourier map
*R*[*F*^2^ > 2σ(*F*^2^)] = 0.043	Hydrogen site location: inferred from neighbouring sites
*wR*(*F*^2^) = 0.138	H atoms treated by a mixture of independent and constrained refinement
*S* = 1.07	*w* = 1/[σ^2^(*F*_o_^2^) + (0.0773*P*)^2^ + 0.6949*P*] where *P* = (*F*_o_^2^ + 2*F*_c_^2^)/3
7179 reflections	(Δ/σ)_max_ < 0.001
598 parameters	Δρ_max_ = 0.72 e Å^−^^3^
4 restraints	Δρ_min_ = −0.32 e Å^−^^3^

### Special details

**Table:**

Geometry. All e.s.d.'s (except the e.s.d. in the dihedral angle between two l.s. planes) are estimated using the full covariance matrix. The cell e.s.d.'s are taken into account individually in the estimation of e.s.d.'s in distances, angles and torsion angles; correlations between e.s.d.'s in cell parameters are only used when they are defined by crystal symmetry. An approximate (isotropic) treatment of cell e.s.d.'s is used for estimating e.s.d.'s involving l.s. planes.
Refinement. Refinement of *F*^2^ against ALL reflections. The weighted *R*-factor *wR* and goodness of fit *S* are based on *F*^2^, conventional *R*-factors *R* are based on *F*, with *F* set to zero for negative *F*^2^. The threshold expression of *F*^2^ > σ(*F*^2^) is used only for calculating *R*-factors(gt) *etc*. and is not relevant to the choice of reflections for refinement. *R*-factors based on *F*^2^ are statistically about twice as large as those based on *F*, and *R*-factors based on ALL data will be even larger.

### Fractional atomic coordinates and isotropic or equivalent isotropic displacement parameters (Å^2^)

**Table:**

	*x*	*y*	*z*	*U*_iso_*/*U*_eq_	Occ. (<1)
C1	0.73604 (16)	0.44594 (17)	−0.05043 (16)	0.0343 (5)	
C2	0.60151 (16)	0.40344 (17)	0.05684 (15)	0.0309 (4)	
C3	0.65908 (16)	0.32478 (16)	0.08526 (15)	0.0307 (4)	
C4	0.75520 (16)	0.30658 (16)	0.04682 (16)	0.0330 (4)	
C5	0.62072 (15)	0.26499 (16)	0.15797 (15)	0.0309 (4)	
C6	0.59379 (16)	0.32162 (17)	0.24693 (16)	0.0331 (4)	
H6	0.6024	0.3968	0.2582	0.040*	
C7	0.55516 (17)	0.27072 (18)	0.31856 (16)	0.0369 (5)	
C8	0.54654 (19)	0.15898 (19)	0.30541 (17)	0.0407 (5)	
C9	0.57638 (18)	0.10095 (19)	0.22112 (18)	0.0423 (5)	
H9	0.5736	0.0267	0.2131	0.051*	
C10	0.61032 (17)	0.15297 (17)	0.14886 (16)	0.0356 (5)	
C11	0.3083 (2)	0.2680 (2)	0.1612 (2)	0.0532 (6)	
H11A	0.3635	0.3322	0.1995	0.064*	
H11B	0.3476	0.2110	0.1388	0.064*	
C12	0.23267 (19)	0.23115 (19)	0.23035 (18)	0.0448 (5)	
H12A	0.2757	0.2063	0.2839	0.054*	
H12B	0.2042	0.2925	0.2629	0.054*	
C13	0.1698 (2)	0.0418 (2)	0.1186 (2)	0.0518 (6)	
H13A	0.1032	−0.0071	0.0756	0.062*	
H13B	0.2191	0.0628	0.0739	0.062*	
C14	0.2266 (3)	−0.0185 (3)	0.1866 (3)	0.0890 (11)	
H14A	0.2944	0.0280	0.2278	0.133*	
H14B	0.2441	−0.0813	0.1456	0.133*	
H14C	0.1781	−0.0414	0.2303	0.133*	
C15	0.0554 (2)	0.1188 (2)	0.2403 (2)	0.0544 (6)	
H15A	0.0367	0.1866	0.2717	0.065*	
H15B	0.0904	0.0894	0.2947	0.065*	
C16	−0.0505 (3)	0.0396 (3)	0.1822 (3)	0.0785 (9)	
H16A	−0.0810	0.0644	0.1236	0.118*	
H16B	−0.1039	0.0350	0.2261	0.118*	
H16C	−0.0341	−0.0309	0.1600	0.118*	
N1	0.78912 (15)	0.37048 (15)	−0.01912 (14)	0.0371 (4)	
N2	0.64328 (14)	0.45918 (15)	−0.01195 (14)	0.0347 (4)	
N3	0.62568 (17)	0.08057 (16)	0.05553 (17)	0.0474 (5)	
N4	0.5033 (2)	0.0960 (2)	0.37474 (18)	0.0619 (6)	
N5	0.13683 (15)	0.14164 (15)	0.17278 (14)	0.0383 (4)	
H5	0.1002	0.1682	0.1228	0.046*	
O1	0.77048 (13)	0.49917 (13)	−0.10940 (13)	0.0454 (4)	
O2	0.81174 (12)	0.24002 (13)	0.06849 (13)	0.0452 (4)	
O3	0.51542 (12)	0.42531 (13)	0.08530 (12)	0.0404 (4)	
O4	0.58787 (16)	0.09712 (15)	−0.02727 (14)	0.0595 (5)	
O5	0.67161 (17)	0.00520 (15)	0.06664 (17)	0.0685 (6)	
O6	0.5307 (3)	0.0114 (2)	0.3777 (2)	0.1097 (10)	
O7	0.4287 (2)	0.12591 (19)	0.41688 (18)	0.0825 (7)	
O8	0.24764 (14)	0.29216 (14)	0.07518 (13)	0.0521 (4)	
H8	0.2158	0.3415	0.0939	0.078*	
O9	1.00189 (14)	0.19327 (14)	0.01618 (15)	0.0494 (4)	
Cl1	0.52193 (6)	0.35212 (5)	0.42199 (5)	0.05531 (19)	
H9A	0.9462 (17)	0.210 (2)	0.040 (2)	0.069 (9)*	
H9B	1.030 (2)	0.241 (2)	−0.013 (3)	0.100 (13)*	
H2	0.604 (2)	0.501 (2)	−0.0340 (18)	0.042 (7)*	
H1	0.847 (2)	0.3646 (18)	−0.0432 (17)	0.034 (6)*	
C17	1.03464 (17)	0.41704 (18)	−0.14752 (16)	0.0368 (5)	
C18	1.16333 (15)	0.44983 (15)	−0.26248 (15)	0.0288 (4)	
C19	1.10360 (16)	0.52326 (16)	−0.29751 (15)	0.0305 (4)	
C20	1.01060 (16)	0.54779 (17)	−0.25484 (15)	0.0324 (4)	
C21	1.13552 (15)	0.57684 (16)	−0.37742 (15)	0.0298 (4)	
C22	1.13816 (17)	0.68886 (17)	−0.36557 (17)	0.0349 (5)	
H22	1.1219	0.7271	−0.3059	0.042*	
C23	1.16361 (19)	0.74390 (17)	−0.43818 (18)	0.0413 (5)	
C24	1.19176 (19)	0.68958 (19)	−0.52631 (18)	0.0438 (5)	
C25	1.19514 (18)	0.58186 (18)	−0.54005 (17)	0.0394 (5)	
H25	1.2168	0.5457	−0.5978	0.047*	
C26	1.16608 (16)	0.52740 (16)	−0.46732 (15)	0.0313 (4)	
C27	1.3675 (3)	0.2472 (3)	−0.1336 (2)	0.0674 (8)	
H27A	1.4013	0.2112	−0.0839	0.081*	
H27B	1.4104	0.3210	−0.1199	0.081*	
C28	1.3701 (3)	0.1885 (3)	−0.2380 (2)	0.0689 (8)	
H28A	1.3201	0.1178	−0.2534	0.083*	
H28B	1.4451	0.1762	−0.2395	0.083*	
C29	1.4321 (2)	0.2946 (2)	−0.3634 (2)	0.0583 (7)	
H29A	1.4687	0.2376	−0.3916	0.070*	
H29B	1.4018	0.3248	−0.4192	0.070*	
C30	1.5159 (3)	0.3808 (3)	−0.2878 (3)	0.0836 (10)	
H30A	1.4789	0.4318	−0.2518	0.125*	
H30B	1.5664	0.4178	−0.3226	0.125*	
H30C	1.5571	0.3489	−0.2399	0.125*	
C31	1.2477 (3)	0.1671 (2)	−0.4101 (2)	0.0680 (8)	
H31A	1.2399	0.2005	−0.4682	0.082*	
H31B	1.2727	0.0997	−0.4309	0.082*	
C32	1.1376 (3)	0.1425 (3)	−0.3805 (3)	0.0822 (10)	
H32A	1.1450	0.1089	−0.3233	0.123*	
H32B	1.0849	0.0938	−0.4374	0.123*	
H32C	1.1117	0.2088	−0.3618	0.123*	
N6	0.98182 (15)	0.49133 (16)	−0.18171 (15)	0.0394 (4)	
N7	1.12560 (14)	0.40106 (15)	−0.18818 (14)	0.0353 (4)	
N8	1.16366 (16)	0.40985 (15)	−0.49451 (13)	0.0381 (4)	
N9	1.2163 (3)	0.7445 (2)	−0.6078 (2)	0.0779 (8)	
N10	1.33782 (18)	0.24545 (16)	−0.31949 (16)	0.0493 (5)	
H10	1.3060	0.3004	−0.2917	0.059*	
O10	1.00397 (14)	0.36887 (15)	−0.08398 (13)	0.0519 (4)	
O11	0.95065 (12)	0.61060 (13)	−0.27829 (12)	0.0418 (4)	
O12	1.24892 (11)	0.42489 (11)	−0.29138 (11)	0.0335 (3)	
O13	1.08502 (14)	0.34754 (13)	−0.48101 (13)	0.0483 (4)	
O14	1.24008 (15)	0.38142 (14)	−0.53307 (13)	0.0555 (5)	
O15	1.1439 (4)	0.7960 (5)	−0.6401 (4)	0.118 (2)	0.670 (9)
O16	1.2847 (6)	0.7283 (7)	−0.6494 (7)	0.142 (5)	0.670 (9)
O15'	1.3101 (9)	0.8218 (9)	−0.5731 (7)	0.125 (5)	0.330 (9)
O16'	1.2011 (14)	0.7054 (10)	−0.6879 (7)	0.133 (8)	0.330 (9)
O17	1.25677 (16)	0.24953 (16)	−0.12333 (14)	0.0623 (5)	
H17	1.2564	0.2834	−0.0655	0.094*	
Cl2	1.16163 (7)	0.88047 (5)	−0.41687 (6)	0.0707 (2)	
H7	1.161 (2)	0.355 (2)	−0.1702 (19)	0.043 (7)*	
H6A	0.928 (2)	0.5042 (19)	−0.1581 (18)	0.036 (6)*	

### Atomic displacement parameters (Å^2^)

**Table:**

	*U*^11^	*U*^22^	*U*^33^	*U*^12^	*U*^13^	*U*^23^
C1	0.0309 (10)	0.0399 (12)	0.0377 (11)	0.0116 (9)	0.0126 (9)	0.0136 (9)
C2	0.0273 (10)	0.0387 (11)	0.0304 (10)	0.0113 (8)	0.0090 (8)	0.0106 (9)
C3	0.0272 (10)	0.0351 (11)	0.0329 (11)	0.0087 (8)	0.0094 (8)	0.0104 (9)
C4	0.0283 (10)	0.0350 (11)	0.0401 (11)	0.0101 (9)	0.0107 (9)	0.0130 (9)
C5	0.0221 (9)	0.0381 (11)	0.0357 (11)	0.0096 (8)	0.0062 (8)	0.0128 (9)
C6	0.0286 (10)	0.0380 (11)	0.0356 (11)	0.0109 (8)	0.0074 (9)	0.0109 (9)
C7	0.0328 (10)	0.0487 (13)	0.0322 (11)	0.0118 (9)	0.0088 (9)	0.0116 (9)
C8	0.0422 (12)	0.0496 (13)	0.0372 (12)	0.0119 (10)	0.0125 (10)	0.0206 (10)
C9	0.0433 (12)	0.0390 (12)	0.0518 (14)	0.0142 (10)	0.0144 (11)	0.0182 (10)
C10	0.0322 (10)	0.0397 (12)	0.0382 (12)	0.0115 (9)	0.0117 (9)	0.0098 (9)
C11	0.0386 (12)	0.0547 (15)	0.0649 (17)	0.0085 (11)	0.0021 (12)	0.0181 (13)
C12	0.0474 (13)	0.0452 (13)	0.0415 (13)	0.0132 (10)	0.0049 (10)	0.0094 (10)
C13	0.0555 (14)	0.0512 (14)	0.0495 (14)	0.0158 (12)	0.0194 (12)	0.0027 (11)
C14	0.101 (3)	0.070 (2)	0.103 (3)	0.051 (2)	0.014 (2)	0.0113 (19)
C15	0.0640 (16)	0.0570 (15)	0.0549 (15)	0.0191 (13)	0.0339 (13)	0.0196 (12)
C16	0.0639 (18)	0.088 (2)	0.088 (2)	−0.0028 (16)	0.0363 (17)	0.0307 (19)
N1	0.0301 (9)	0.0450 (11)	0.0484 (11)	0.0180 (8)	0.0207 (8)	0.0201 (9)
N2	0.0307 (9)	0.0432 (10)	0.0410 (10)	0.0189 (8)	0.0147 (8)	0.0196 (8)
N3	0.0462 (11)	0.0405 (11)	0.0575 (14)	0.0067 (9)	0.0253 (10)	0.0060 (10)
N4	0.0846 (17)	0.0630 (15)	0.0533 (13)	0.0228 (13)	0.0316 (12)	0.0280 (11)
N5	0.0423 (10)	0.0432 (10)	0.0362 (10)	0.0150 (8)	0.0146 (8)	0.0148 (8)
O1	0.0426 (8)	0.0569 (10)	0.0557 (10)	0.0232 (7)	0.0266 (8)	0.0332 (8)
O2	0.0372 (8)	0.0490 (9)	0.0673 (11)	0.0240 (7)	0.0249 (8)	0.0309 (8)
O3	0.0340 (8)	0.0566 (10)	0.0459 (9)	0.0254 (7)	0.0198 (7)	0.0246 (7)
O4	0.0692 (12)	0.0605 (12)	0.0431 (10)	0.0026 (9)	0.0183 (9)	0.0026 (9)
O5	0.0776 (13)	0.0476 (11)	0.0946 (15)	0.0287 (10)	0.0454 (12)	0.0139 (10)
O6	0.166 (3)	0.0938 (18)	0.129 (2)	0.0676 (19)	0.090 (2)	0.0805 (18)
O7	0.1039 (17)	0.0817 (15)	0.0758 (14)	0.0151 (13)	0.0538 (13)	0.0256 (12)
O8	0.0519 (10)	0.0570 (10)	0.0532 (10)	0.0116 (8)	0.0154 (8)	0.0223 (8)
O9	0.0389 (9)	0.0554 (10)	0.0675 (12)	0.0219 (8)	0.0200 (8)	0.0280 (9)
Cl1	0.0693 (4)	0.0612 (4)	0.0400 (3)	0.0161 (3)	0.0252 (3)	0.0086 (3)
C17	0.0343 (11)	0.0450 (12)	0.0384 (12)	0.0144 (9)	0.0127 (9)	0.0169 (10)
C18	0.0287 (10)	0.0291 (10)	0.0292 (10)	0.0074 (8)	0.0068 (8)	0.0059 (8)
C19	0.0308 (10)	0.0320 (10)	0.0326 (10)	0.0107 (8)	0.0094 (8)	0.0103 (8)
C20	0.0315 (10)	0.0349 (11)	0.0338 (11)	0.0121 (9)	0.0078 (9)	0.0096 (9)
C21	0.0237 (9)	0.0319 (10)	0.0345 (11)	0.0083 (8)	0.0041 (8)	0.0082 (8)
C22	0.0342 (11)	0.0327 (11)	0.0408 (12)	0.0101 (9)	0.0106 (9)	0.0101 (9)
C23	0.0428 (12)	0.0297 (11)	0.0535 (14)	0.0083 (9)	0.0098 (10)	0.0140 (10)
C24	0.0460 (13)	0.0455 (13)	0.0468 (13)	0.0073 (10)	0.0150 (11)	0.0241 (11)
C25	0.0381 (11)	0.0443 (13)	0.0388 (12)	0.0096 (10)	0.0124 (10)	0.0117 (10)
C26	0.0300 (10)	0.0309 (10)	0.0345 (11)	0.0084 (8)	0.0067 (8)	0.0096 (9)
C27	0.0680 (18)	0.088 (2)	0.0527 (16)	0.0331 (16)	0.0112 (14)	0.0186 (15)
C28	0.086 (2)	0.0715 (19)	0.0711 (19)	0.0483 (17)	0.0276 (16)	0.0301 (16)
C29	0.0615 (16)	0.0704 (18)	0.0538 (16)	0.0268 (14)	0.0279 (13)	0.0156 (13)
C30	0.0648 (19)	0.096 (3)	0.080 (2)	0.0135 (18)	0.0264 (17)	−0.0080 (19)
C31	0.094 (2)	0.0429 (15)	0.0690 (19)	0.0187 (15)	0.0204 (17)	0.0095 (13)
C32	0.081 (2)	0.0540 (18)	0.103 (3)	0.0091 (16)	0.002 (2)	0.0162 (18)
N6	0.0351 (10)	0.0512 (12)	0.0476 (11)	0.0238 (9)	0.0221 (9)	0.0245 (9)
N7	0.0353 (9)	0.0413 (10)	0.0408 (10)	0.0191 (8)	0.0156 (8)	0.0204 (8)
N8	0.0470 (11)	0.0368 (10)	0.0306 (9)	0.0148 (9)	0.0029 (8)	0.0070 (8)
N9	0.118 (2)	0.0601 (16)	0.078 (2)	0.0223 (17)	0.0525 (19)	0.0383 (16)
N10	0.0656 (13)	0.0449 (11)	0.0542 (12)	0.0313 (10)	0.0286 (10)	0.0203 (10)
O10	0.0513 (9)	0.0700 (11)	0.0608 (11)	0.0309 (9)	0.0327 (8)	0.0438 (9)
O11	0.0402 (8)	0.0490 (9)	0.0500 (9)	0.0259 (7)	0.0164 (7)	0.0231 (7)
O12	0.0322 (7)	0.0352 (8)	0.0412 (8)	0.0154 (6)	0.0153 (6)	0.0138 (6)
O13	0.0550 (10)	0.0349 (8)	0.0498 (10)	0.0011 (7)	0.0026 (8)	0.0108 (7)
O14	0.0700 (11)	0.0579 (11)	0.0498 (10)	0.0366 (9)	0.0221 (9)	0.0103 (8)
O15	0.149 (4)	0.155 (5)	0.118 (4)	0.090 (3)	0.066 (3)	0.110 (4)
O16	0.160 (6)	0.184 (9)	0.188 (9)	0.113 (7)	0.139 (7)	0.141 (8)
O15'	0.154 (9)	0.103 (7)	0.093 (6)	−0.068 (6)	0.040 (6)	0.032 (6)
O16'	0.221 (16)	0.107 (9)	0.039 (5)	−0.058 (11)	0.027 (7)	0.020 (4)
O17	0.0757 (13)	0.0789 (13)	0.0519 (11)	0.0432 (11)	0.0218 (9)	0.0299 (10)
Cl2	0.0971 (6)	0.0333 (3)	0.0919 (6)	0.0188 (3)	0.0292 (4)	0.0254 (3)

### Geometric parameters (Å, °)

**Table:**

C1—O1	1.231 (2)	C17—O10	1.228 (2)
C1—N1	1.355 (3)	C17—N6	1.356 (3)
C1—N2	1.357 (3)	C17—N7	1.358 (3)
C2—O3	1.246 (2)	C18—O12	1.255 (2)
C2—N2	1.395 (3)	C18—N7	1.389 (3)
C2—C3	1.408 (3)	C18—C19	1.400 (3)
C3—C4	1.409 (3)	C19—C20	1.422 (3)
C3—C5	1.454 (3)	C19—C21	1.465 (3)
C4—O2	1.241 (2)	C20—O11	1.236 (2)
C4—N1	1.394 (3)	C20—N6	1.396 (3)
C5—C10	1.398 (3)	C21—C26	1.398 (3)
C5—C6	1.399 (3)	C21—C22	1.406 (3)
C6—C7	1.377 (3)	C22—C23	1.368 (3)
C6—H6	0.9300	C22—H22	0.9300
C7—C8	1.390 (3)	C23—C24	1.386 (3)
C7—Cl1	1.725 (2)	C23—Cl2	1.721 (2)
C8—C9	1.374 (3)	C24—C25	1.364 (3)
C8—N4	1.464 (3)	C24—N9	1.468 (3)
C9—C10	1.372 (3)	C25—C26	1.377 (3)
C9—H9	0.9300	C25—H25	0.9300
C10—N3	1.473 (3)	C26—N8	1.471 (3)
C11—O8	1.403 (3)	C27—O17	1.404 (3)
C11—C12	1.512 (3)	C27—C28	1.476 (4)
C11—H11A	0.9700	C27—H27A	0.9700
C11—H11B	0.9700	C27—H27B	0.9700
C12—N5	1.491 (3)	C28—N10	1.482 (3)
C12—H12A	0.9700	C28—H28A	0.9700
C12—H12B	0.9700	C28—H28B	0.9700
C13—C14	1.486 (4)	C29—C30	1.474 (4)
C13—N5	1.497 (3)	C29—N10	1.495 (3)
C13—H13A	0.9700	C29—H29A	0.9700
C13—H13B	0.9700	C29—H29B	0.9700
C14—H14A	0.9600	C30—H30A	0.9600
C14—H14B	0.9600	C30—H30B	0.9600
C14—H14C	0.9600	C30—H30C	0.9600
C15—N5	1.498 (3)	C31—C32	1.482 (5)
C15—C16	1.508 (4)	C31—N10	1.568 (4)
C15—H15A	0.9700	C31—H31A	0.9700
C15—H15B	0.9700	C31—H31B	0.9700
C16—H16A	0.9600	C32—H32A	0.9600
C16—H16B	0.9600	C32—H32B	0.9600
C16—H16C	0.9600	C32—H32C	0.9600
N1—H1	0.84 (2)	N6—H6A	0.81 (2)
N2—H2	0.85 (3)	N7—H7	0.84 (3)
N3—O4	1.221 (3)	N8—O13	1.213 (2)
N3—O5	1.223 (3)	N8—O14	1.225 (2)
N4—O6	1.194 (3)	N9—O16'	1.076 (9)
N4—O7	1.235 (3)	N9—O16	1.115 (5)
N5—H5	0.9100	N9—O15	1.275 (5)
O8—H8	0.8200	N9—O15'	1.343 (8)
O9—H9A	0.844 (10)	N10—H10	0.9100
O9—H9B	0.847 (10)	O17—H17	0.8200
			
O1—C1—N1	121.94 (18)	O12—C18—N7	116.88 (17)
O1—C1—N2	122.51 (19)	O12—C18—C19	125.81 (18)
N1—C1—N2	115.54 (18)	N7—C18—C19	117.30 (17)
O3—C2—N2	117.45 (17)	C18—C19—C20	120.00 (18)
O3—C2—C3	125.94 (18)	C18—C19—C21	121.25 (17)
N2—C2—C3	116.58 (16)	C20—C19—C21	118.75 (17)
C2—C3—C4	120.63 (18)	O11—C20—N6	116.96 (17)
C2—C3—C5	119.26 (17)	O11—C20—C19	126.90 (19)
C4—C3—C5	120.07 (17)	N6—C20—C19	116.09 (17)
O2—C4—N1	118.45 (17)	C26—C21—C22	114.50 (18)
O2—C4—C3	125.05 (19)	C26—C21—C19	126.22 (17)
N1—C4—C3	116.49 (17)	C22—C21—C19	119.28 (17)
C10—C5—C6	115.15 (18)	C23—C22—C21	122.7 (2)
C10—C5—C3	125.52 (18)	C23—C22—H22	118.6
C6—C5—C3	119.31 (18)	C21—C22—H22	118.6
C7—C6—C5	122.8 (2)	C22—C23—C24	119.8 (2)
C7—C6—H6	118.6	C22—C23—Cl2	118.85 (18)
C5—C6—H6	118.6	C24—C23—Cl2	121.37 (17)
C6—C7—C8	119.43 (19)	C25—C24—C23	120.1 (2)
C6—C7—Cl1	116.87 (17)	C25—C24—N9	118.2 (2)
C8—C7—Cl1	123.70 (16)	C23—C24—N9	121.7 (2)
C9—C8—C7	119.59 (19)	C24—C25—C26	119.1 (2)
C9—C8—N4	116.3 (2)	C24—C25—H25	120.5
C7—C8—N4	124.1 (2)	C26—C25—H25	120.5
C10—C9—C8	119.8 (2)	C25—C26—C21	123.73 (19)
C10—C9—H9	120.1	C25—C26—N8	114.20 (18)
C8—C9—H9	120.1	C21—C26—N8	121.99 (17)
C9—C10—C5	123.18 (19)	O17—C27—C28	110.3 (3)
C9—C10—N3	114.93 (19)	O17—C27—H27A	109.6
C5—C10—N3	121.68 (18)	C28—C27—H27A	109.6
O8—C11—C12	111.63 (19)	O17—C27—H27B	109.6
O8—C11—H11A	109.3	C28—C27—H27B	109.6
C12—C11—H11A	109.3	H27A—C27—H27B	108.1
O8—C11—H11B	109.3	C27—C28—N10	114.6 (2)
C12—C11—H11B	109.3	C27—C28—H28A	108.6
H11A—C11—H11B	108.0	N10—C28—H28A	108.6
N5—C12—C11	111.96 (19)	C27—C28—H28B	108.6
N5—C12—H12A	109.2	N10—C28—H28B	108.6
C11—C12—H12A	109.2	H28A—C28—H28B	107.6
N5—C12—H12B	109.2	C30—C29—N10	113.5 (2)
C11—C12—H12B	109.2	C30—C29—H29A	108.9
H12A—C12—H12B	107.9	N10—C29—H29A	108.9
C14—C13—N5	115.1 (2)	C30—C29—H29B	108.9
C14—C13—H13A	108.5	N10—C29—H29B	108.9
N5—C13—H13A	108.5	H29A—C29—H29B	107.7
C14—C13—H13B	108.5	C29—C30—H30A	109.5
N5—C13—H13B	108.5	C29—C30—H30B	109.5
H13A—C13—H13B	107.5	H30A—C30—H30B	109.5
C13—C14—H14A	109.5	C29—C30—H30C	109.5
C13—C14—H14B	109.5	H30A—C30—H30C	109.5
H14A—C14—H14B	109.5	H30B—C30—H30C	109.5
C13—C14—H14C	109.5	C32—C31—N10	111.7 (2)
H14A—C14—H14C	109.5	C32—C31—H31A	109.3
H14B—C14—H14C	109.5	N10—C31—H31A	109.3
N5—C15—C16	112.4 (2)	C32—C31—H31B	109.3
N5—C15—H15A	109.1	N10—C31—H31B	109.3
C16—C15—H15A	109.1	H31A—C31—H31B	107.9
N5—C15—H15B	109.1	C31—C32—H32A	109.5
C16—C15—H15B	109.1	C31—C32—H32B	109.5
H15A—C15—H15B	107.9	H32A—C32—H32B	109.5
C15—C16—H16A	109.5	C31—C32—H32C	109.5
C15—C16—H16B	109.5	H32A—C32—H32C	109.5
H16A—C16—H16B	109.5	H32B—C32—H32C	109.5
C15—C16—H16C	109.5	C17—N6—C20	126.19 (18)
H16A—C16—H16C	109.5	C17—N6—H6A	117.4 (17)
H16B—C16—H16C	109.5	C20—N6—H6A	116.4 (17)
C1—N1—C4	125.43 (17)	C17—N7—C18	125.73 (18)
C1—N1—H1	113.6 (15)	C17—N7—H7	118.2 (17)
C4—N1—H1	120.9 (15)	C18—N7—H7	115.9 (17)
C1—N2—C2	125.29 (18)	O13—N8—O14	124.30 (19)
C1—N2—H2	118.9 (16)	O13—N8—C26	118.52 (17)
C2—N2—H2	115.6 (16)	O14—N8—C26	117.12 (18)
O4—N3—O5	124.6 (2)	O16'—N9—O16	57.1 (8)
O4—N3—C10	118.05 (19)	O16'—N9—O15	81.8 (10)
O5—N3—C10	117.3 (2)	O16—N9—O15	121.3 (4)
O6—N4—O7	122.9 (2)	O16'—N9—O15'	115.5 (8)
O6—N4—C8	118.8 (2)	O16—N9—O15'	67.1 (7)
O7—N4—C8	117.6 (2)	O15—N9—O15'	103.5 (7)
C12—N5—C13	114.51 (18)	O16'—N9—C24	125.0 (6)
C12—N5—C15	110.22 (18)	O16—N9—C24	121.6 (4)
C13—N5—C15	113.58 (18)	O15—N9—C24	116.0 (3)
C12—N5—H5	105.9	O15'—N9—C24	110.0 (4)
C13—N5—H5	105.9	C28—N10—C29	115.2 (2)
C15—N5—H5	105.9	C28—N10—C31	110.8 (2)
C11—O8—H8	109.5	C29—N10—C31	108.0 (2)
H9A—O9—H9B	111.6 (17)	C28—N10—H10	107.5
O10—C17—N6	122.98 (19)	C29—N10—H10	107.5
O10—C17—N7	122.42 (19)	C31—N10—H10	107.5
N6—C17—N7	114.59 (18)	C27—O17—H17	109.5
			
O3—C2—C3—C4	179.2 (2)	O12—C18—C19—C21	2.3 (3)
N2—C2—C3—C4	1.3 (3)	N7—C18—C19—C21	−178.60 (18)
O3—C2—C3—C5	−3.0 (3)	C18—C19—C20—O11	−180.0 (2)
N2—C2—C3—C5	179.08 (18)	C21—C19—C20—O11	0.5 (3)
C2—C3—C4—O2	179.2 (2)	C18—C19—C20—N6	−2.7 (3)
C5—C3—C4—O2	1.5 (3)	C21—C19—C20—N6	177.79 (18)
C2—C3—C4—N1	0.4 (3)	C18—C19—C21—C26	43.5 (3)
C5—C3—C4—N1	−177.35 (18)	C20—C19—C21—C26	−137.0 (2)
C2—C3—C5—C10	136.1 (2)	C18—C19—C21—C22	−135.7 (2)
C4—C3—C5—C10	−46.2 (3)	C20—C19—C21—C22	43.7 (3)
C2—C3—C5—C6	−45.7 (3)	C26—C21—C22—C23	2.8 (3)
C4—C3—C5—C6	132.0 (2)	C19—C21—C22—C23	−177.86 (19)
C10—C5—C6—C7	−2.7 (3)	C21—C22—C23—C24	−2.1 (3)
C3—C5—C6—C7	178.89 (18)	C21—C22—C23—Cl2	178.70 (16)
C5—C6—C7—C8	2.8 (3)	C22—C23—C24—C25	−0.6 (3)
C5—C6—C7—Cl1	−178.12 (15)	Cl2—C23—C24—C25	178.48 (18)
C6—C7—C8—C9	−0.2 (3)	C22—C23—C24—N9	178.1 (3)
Cl1—C7—C8—C9	−179.13 (17)	Cl2—C23—C24—N9	−2.8 (3)
C6—C7—C8—N4	−178.0 (2)	C23—C24—C25—C26	2.5 (3)
Cl1—C7—C8—N4	3.1 (3)	N9—C24—C25—C26	−176.3 (2)
C7—C8—C9—C10	−2.5 (3)	C24—C25—C26—C21	−1.7 (3)
N4—C8—C9—C10	175.5 (2)	C24—C25—C26—N8	175.07 (19)
C8—C9—C10—C5	2.6 (3)	C22—C21—C26—C25	−0.9 (3)
C8—C9—C10—N3	−172.2 (2)	C19—C21—C26—C25	179.86 (19)
C6—C5—C10—C9	0.0 (3)	C22—C21—C26—N8	−177.43 (17)
C3—C5—C10—C9	178.3 (2)	C19—C21—C26—N8	3.3 (3)
C6—C5—C10—N3	174.38 (18)	O17—C27—C28—N10	68.8 (4)
C3—C5—C10—N3	−7.3 (3)	O10—C17—N6—C20	−178.8 (2)
O8—C11—C12—N5	51.5 (3)	N7—C17—N6—C20	2.0 (3)
O1—C1—N1—C4	−179.6 (2)	O11—C20—N6—C17	178.2 (2)
N2—C1—N1—C4	0.8 (3)	C19—C20—N6—C17	0.7 (3)
O2—C4—N1—C1	179.5 (2)	O10—C17—N7—C18	177.9 (2)
C3—C4—N1—C1	−1.6 (3)	N6—C17—N7—C18	−3.0 (3)
O1—C1—N2—C2	−178.4 (2)	O12—C18—N7—C17	−179.7 (2)
N1—C1—N2—C2	1.2 (3)	C19—C18—N7—C17	1.1 (3)
O3—C2—N2—C1	179.7 (2)	C25—C26—N8—O13	−134.9 (2)
C3—C2—N2—C1	−2.2 (3)	C21—C26—N8—O13	42.0 (3)
C9—C10—N3—O4	135.0 (2)	C25—C26—N8—O14	42.5 (2)
C5—C10—N3—O4	−39.9 (3)	C21—C26—N8—O14	−140.6 (2)
C9—C10—N3—O5	−42.1 (3)	C25—C24—N9—O16'	29.0 (14)
C5—C10—N3—O5	143.1 (2)	C23—C24—N9—O16'	−149.7 (13)
C9—C8—N4—O6	25.7 (4)	C25—C24—N9—O16	−40.6 (8)
C7—C8—N4—O6	−156.4 (3)	C23—C24—N9—O16	140.7 (8)
C9—C8—N4—O7	−144.8 (3)	C25—C24—N9—O15	127.6 (4)
C7—C8—N4—O7	33.1 (4)	C23—C24—N9—O15	−51.2 (5)
C11—C12—N5—C13	58.8 (3)	C25—C24—N9—O15'	−115.3 (8)
C11—C12—N5—C15	−171.7 (2)	C23—C24—N9—O15'	65.9 (8)
C14—C13—N5—C12	66.5 (3)	C27—C28—N10—C29	106.6 (3)
C14—C13—N5—C15	−61.3 (3)	C27—C28—N10—C31	−130.3 (3)
C16—C15—N5—C12	172.3 (2)	C30—C29—N10—C28	−62.9 (3)
C16—C15—N5—C13	−57.7 (3)	C30—C29—N10—C31	172.6 (3)
O12—C18—C19—C20	−177.21 (19)	C32—C31—N10—C28	71.2 (3)
N7—C18—C19—C20	1.9 (3)	C32—C31—N10—C29	−161.6 (2)

### Hydrogen-bond geometry (Å, °)

**Table:**

*D*—H···*A*	*D*—H	H···*A*	*D*···*A*	*D*—H···*A*
N5—H5···O9^i^	0.91	1.85	2.749 (2)	167.
O8—H8···O1^ii^	0.82	1.98	2.674 (2)	141.
O9—H9A···O2	0.84 (1)	1.85 (1)	2.691 (2)	171 (3)
O9—H9B···O10	0.85 (1)	2.11 (2)	2.850 (2)	146 (3)
N2—H2···O3^ii^	0.85 (3)	1.99 (3)	2.826 (2)	168 (2)
N1—H1···O10	0.84 (2)	2.10 (2)	2.933 (2)	171 (2)
N10—H10···O12	0.91	1.85	2.698 (2)	154.
O17—H17···O8^iii^	0.82	1.91	2.667 (2)	153.
N7—H7···O17	0.84 (3)	2.07 (3)	2.911 (2)	176 (2)
N6—H6A···O1	0.81 (2)	2.16 (2)	2.947 (2)	167 (2)

Symmetry codes: (i) *x*−1, *y*, *z*; (ii) −*x*+1, −*y*+1, −*z*; (iii) *x*+1, *y*, *z*.
